# A Case of Post-transplant Lymphoproliferative Disorder After Successful Treatment of Islet Transplantation Without Graft Rejection

**DOI:** 10.7759/cureus.85478

**Published:** 2025-06-06

**Authors:** Koki Kurahashi, Takayuki Anazawa, Kei Yamane, Kentaro Tsuji, Tadahiko Matsumoto, Junji Fujikura, Etsuro Hatano

**Affiliations:** 1 Department of Surgery, Kyoto University, Kyoto, JPN; 2 Department of Diagnostic Pathology, Kyoto University, Kyoto, JPN; 3 Department of Hematology, Kyoto University, Kyoto, JPN; 4 Department of Diabetes, Endocrinology and Nutrition, Kyoto University, Kyoto, JPN

**Keywords:** da-epoch-r, graft rejection, immuno suppresion, islet transplant, post transplant lymphoproliferative disorder (ptld)

## Abstract

Post-transplant lymphoproliferative disorder (PTLD) is a lymphoid or plasmacytic proliferation that occurs in association with immunosuppression and is recognized as a relatively rare but serious complication following solid organ transplantation; however, PTLD following islet transplantation is extremely rare, and its optimal management remains unclear. We report the case of a 55-year-old woman with a history of type 1 diabetes mellitus who underwent living-donor kidney transplantation followed by deceased-donor pancreas transplantation and subsequently received three islet transplantations due to recurrent diabetes. Five years after the final islet transplantation, she developed persistent fever, and fluorodeoxyglucose-positron emission tomography/computed tomography (FDG-PET/CT) revealed small intestinal wall thickening and lymphadenopathy in the mesentery and axilla. A biopsy confirmed the diagnosis of diffuse large B-cell lymphoma (DLBCL)-type PTLD. Immunosuppressive therapy was carefully adjusted without complete discontinuation, and initial treatment with four cycles of R-CHOP (rituximab, cyclophosphamide, doxorubicin, vincristine, and prednisone) achieved partial remission. This was followed by four cycles of DA-EPOCH-R (dose-adjusted toposide, prednisone, vincristine, cyclophosphamide, hydroxydaunorubicin, and rituximab), which led to complete remission of PTLD. Notably, islet graft function was preserved throughout treatment, and no recurrence of PTLD was observed during follow-up. To our knowledge, this is the first reported case of PTLD following islet transplantation in Japan, and it highlights the potential for achieving remission with preserved graft function through careful immunosuppressive adjustment and appropriate chemotherapy.

## Introduction

Islet transplantation has been increasingly recognized as a promising therapeutic option for patients with type 1 diabetes who suffer from recurrent severe hypoglycemic events (SHEs). It improves glycemic variability and restores endogenous insulin secretion, thereby contributing to the prevention of SHEs and enhancement of the quality of life [1]. However, long-term graft survival and function depend heavily on immunosuppressive therapy, which poses significant clinical challenges including an increased risk of infection and malignancy. Post-transplant lymphoproliferative disorders (PTLD) are a group of lymphoproliferative and plasma cell disorders that develop mostly by EBV reactivation under immunosuppressive conditions after transplantation. They represent one of the most serious complications following solid organ transplantation, with reported incidence rates of approximately 1-5% in kidney, liver, and heart transplant recipients [2]. Conversely, the incidence of PTLD following islet transplantation is extremely rare.

Reduction or discontinuation of immunosuppressive agents is the first-line treatment for PTLD. However, this approach carries a substantial risk of islet graft rejection or functional loss. Therefore, the treatment of PTLD while preserving islet graft function remains a complex and clinically significant challenge. Herein, we report a rare case of PTLD following islet transplantation in which appropriate adjustment of immunosuppressive therapy combined with chemotherapy led to complete remission while maintaining graft function, along with a review of the literature.

## Case presentation

A woman in her 50s with a long-standing history of type 1 diabetes mellitus diagnosed in adolescence had been receiving insulin therapy since diagnosis. She underwent living-donor kidney transplantation at the age of 38 years, followed by deceased-donor pancreas transplantation at the age of 43 years. However, the pancreatic graft eventually failed because of the recurrence of type 1 diabetes. Following the failure of her pancreatic graft, she experienced recurrent SHEs and opted for islet transplantation. She subsequently underwent three sequential islet transplantations in her 50s.

Prior to the initial islet transplantation, the patient was seropositive for Epstein-Barr virus (EBV) viral capsid antigen (VCA)-IgG and cytomegalovirus (CMV)-IgG, indicating prior exposure to both viruses. At that time, she was receiving maintenance immunosuppressive therapy with tacrolimus and mycophenolate mofetil (MMF), following her previous kidney transplantation. For islet transplantation, induction immunotherapy consisted of antithymocyte globulin (ATG) for the first and third transplants and basiliximab for the second transplant. Maintenance immunosuppression following islet transplantation included tacrolimus (target trough level, 5-8 ng/mL) and MMF (500 mg/day) (Table 1).

**Table 1:** Islet transplantation. MMF, mycophenolate mofetil; PTLD, post-transplant lymphoproliferative disorders; rATG, rabbit anti-human thymocyte globulin

	Date (before PTLD)	Hba1c: % (Pre→1month)	C-peptide: ng/ml (Pre→1month)	Insulin: U/day (Pre→1month)	Islet mass: IE/kg	Induction immunosuppression	Maintenance immunosuppression
1st	5 years 5 months before	7.7→7.0	0.05→0.39	14.7→14	6366	rATG:6.0mg/kg	Tacrolimus(target:5-8ng/ml)
						Etanercept:100mg/kg	MMF250mg/250mg
2nd	4 years 8 months before	7.3→6.5	0.05→1	14→8	9933	Basiliximab:6.0mg/kg	Tacrolimus(target:5-8ng/ml)
						Etanercept:100mg/kg	MMF250mg/250mg
3rd	10 months before	6.7→5.3	0.38→0.89	9→7.5	6366	rATG:6.0mg/kg	Tacrolimus(target:5-8ng/ml)
							MMF250mg/250mg

Her glycemic control before islet transplantation was suboptimal, with an HbA1c level of 7.7%, and she experienced recurrent SHEs more than once per month. After the second islet transplantation, her HbA1c level improved to 5.9% at three months postoperatively, with a reduced daily insulin requirement of 7.5 units and resolution of SHEs. Following the third transplantation, glycemic control further improved, with an HbA1c level of 5.3% and the insulin dose reduced to 7 units/day. Renal graft function remained stable throughout this period.

Ten months after the third islet transplantation, the patient developed a persistent fever exceeding 38°C without an identifiable source and was admitted for further evaluation. At presentation, she maintained good glycemic control and stable renal graft function, with an estimated glomerular filtration rate (eGFR) of 34.1 mL/min/1.73 m² (reference range: >60) and a fasting C-peptide level of 1.2 ng/mL (reference range: 0.5-2.0).

**Figure 1:**
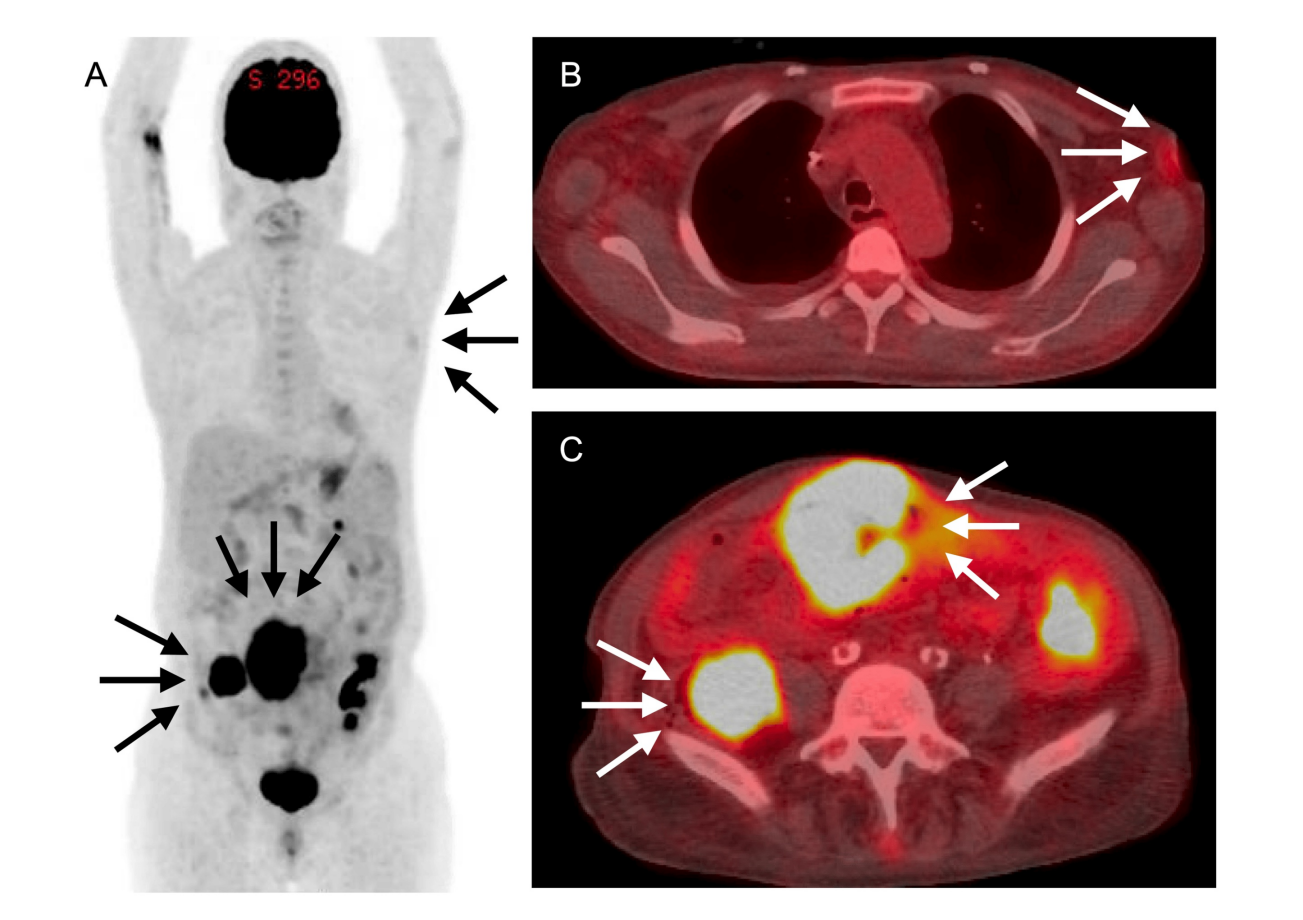
PET-CT findings. (A) Coronal section and (B) axial section of the thorax show FDG uptake in a left axillary mass. (C) The axial abdominal section shows intense FDG uptake in the thickened small intestinal wall immediately beneath the abdominal wall (SUVmax 17.4) and in the right retroperitoneal mass (SUVmax 10.0).

Laboratory tests on admission revealed a white blood cell count of 6,660 /μL (reference range: 4,000-10,000), a C-reactive protein (CRP) level of 16.1 mg/dL (reference range: <0.3), total protein 5.2 g/dL (reference range: 6.6-8.1), albumin 1.9 g/dL (reference range: 4.0-5.0), BUN 38 mg/dL (reference range: 8-20), creatinine 1.3 mg/dL (reference range: 0.6-1.0), and blood glucose 170 mg/dL (reference range: 70-110 fasting). Quantitative EBV-DNA testing of peripheral blood showed 230 copies/μg DNA. Abdominal computed tomography (CT) revealed a 4-cm soft tissue mass adjacent to the right psoas muscle, along with thickening of the small intestinal wall in the mid-abdomen, without significant luminal narrowing, but with associated dilatation. Fluorodeoxyglucose-positron emission tomography/computed tomography (FDG-PET/CT) revealed a hypermetabolic mass in the right retroperitoneal space, with a maximum standardized uptake value (SUVmax) of 10.0. In addition, intense FDG uptake was observed in the thickened intestinal wall (SUV, 17.4), multiple mesenteric lymph nodes, and left axillary lymph nodes (Figure 1).

**Figure 2:**
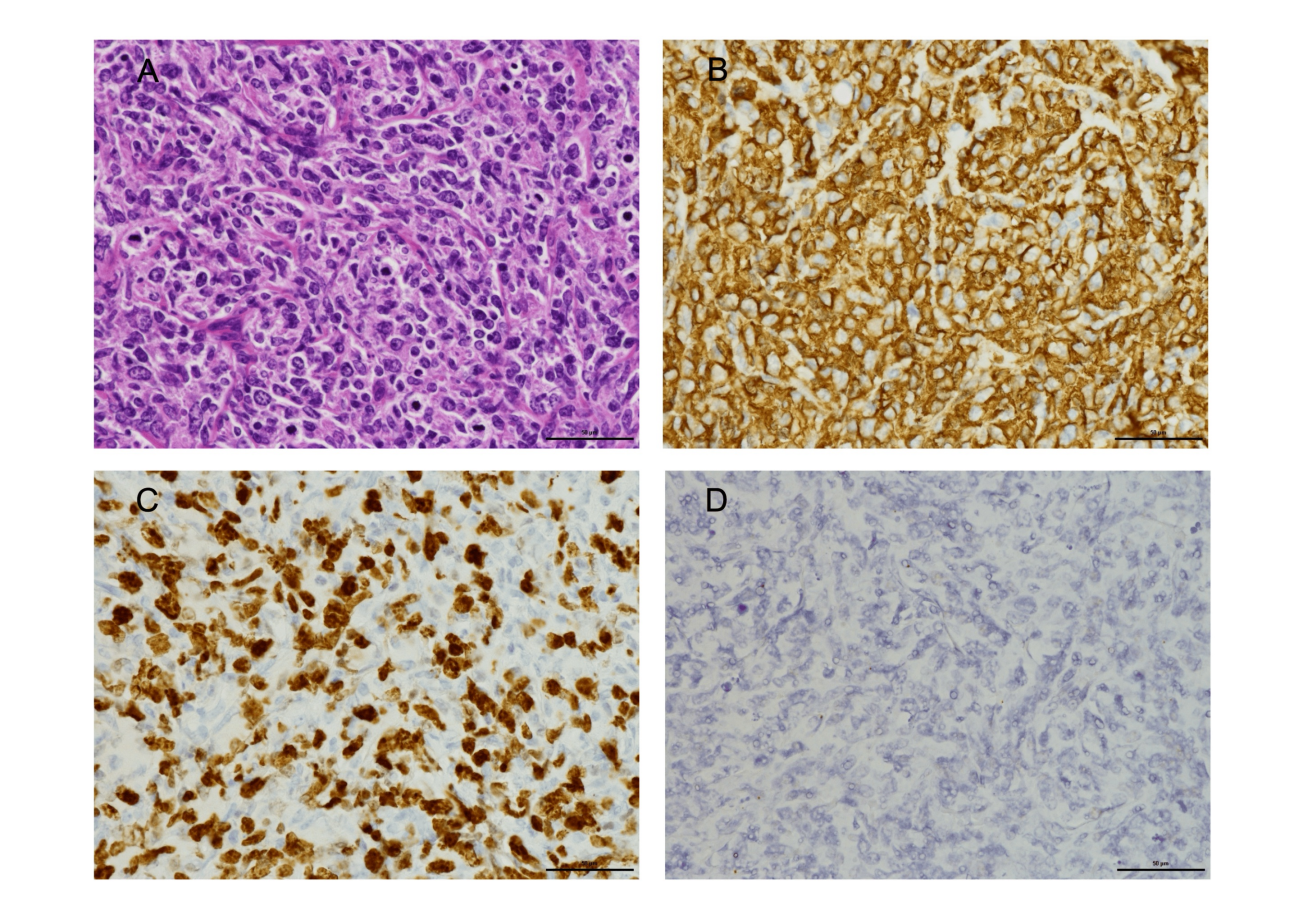
Histopathological findings. (A) Hematoxylin and eosin staining (×400): Diffuse proliferation of large lymphoid cells with vesicular chromatin and multiple small nucleoli. (B) CD20 immunohistochemistry (×400): diffuse positivity. (C) Ki-67 immunohistochemistry (×400): high proliferative activity with a Ki-67 index of approximately 70%. (D) In situ hybridization for Epstein-Barr virus-encoded small RNA (EBER) (×400): negative.

Given these findings, PTLD and infectious diseases were considered in the differential diagnoses. Excisional biopsy of the left axillary lymph node was performed. Histological examination revealed diffuse proliferation of large lymphoid cells with vesicular chromatin and multiple nucleoli on hematoxylin and eosin staining. Immunohistochemistry revealed a strong CD20 positivity and a Ki-67 proliferation index of approximately 70%. In situ hybridization for EBV-encoded RNA (EBER) was negative (Figure 2). Based on these findings, a diagnosis of PTLD consistent with diffuse large B-cell lymphoma (DLBCL) was established.

**Figure 3:**
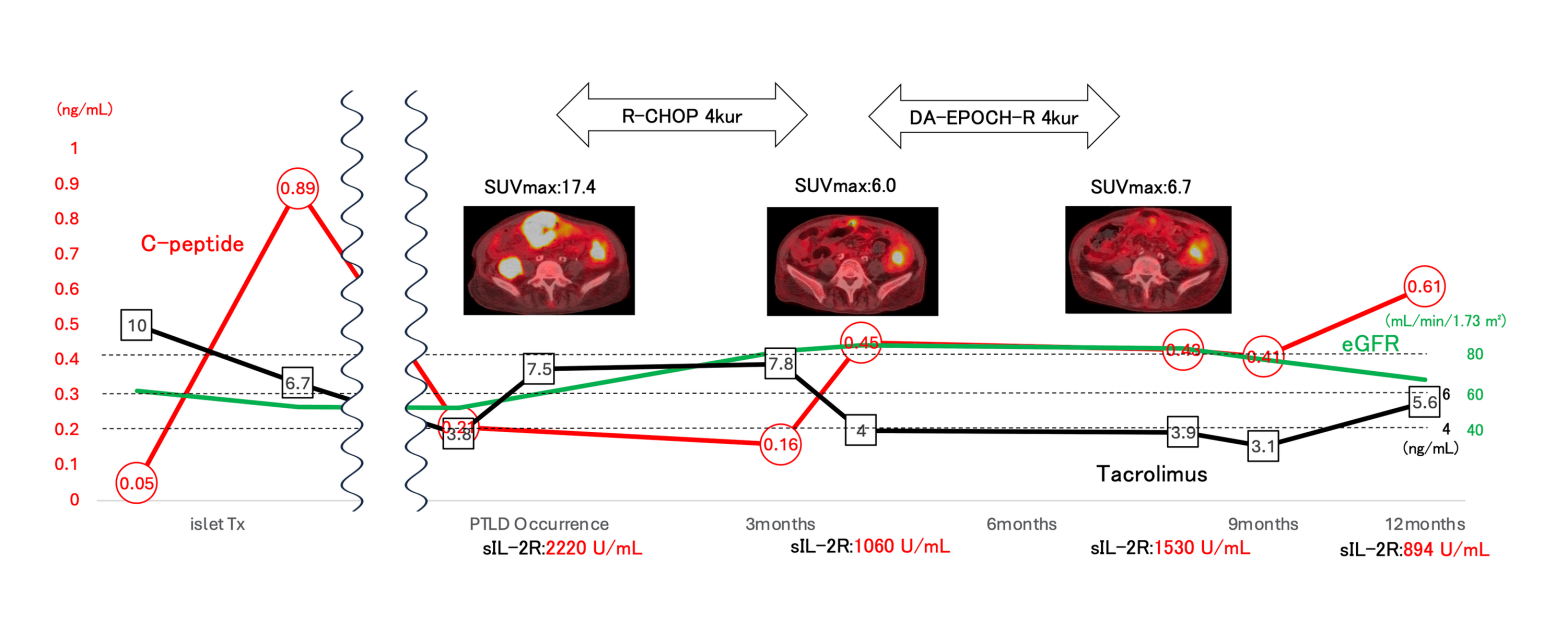
Clinical course and treatment. DA-EPOCH-R, dose-adjusted etoposide, prednisone, vincristine, cyclophosphamide, doxorubicin, and rituximab; PTLD, posttransplant lymphoproliferative disorders; sIL-2R, soluble interleukin-2 receptor; tacrolimus, a calcineurin inhibitor; R-CHOP, rituximab, cyclophosphamide, doxorubicin, vincristine, and prednisone.

Chemotherapy was initiated, while immunosuppressive therapy was continued to maintain renal and islet graft function, although the target trough level of tacrolimus was reduced to approximately 3-6 ng/mL. The patient received four cycles of R-CHOP (rituximab, cyclophosphamide, hydroxydaunorubicin, vincristine, and prednisone). Post-treatment imaging revealed residual masses with persistent FDG uptake, indicating partial remission. Then she received four cycles of DA-EPOCH-R (dose-adjusted etoposide, prednisone, vincristine, cyclophosphamide, hydroxydaunorubicin, and rituximab) as a salvage therapy. FDG-PET/CT performed after therapy completion still showed mild FDG uptake in the lower abdominal wall; however, subsequent contrast-enhanced CT revealed no disease progression, and the uptake was attributed to chronic inflammation. Therefore, the patient was assessed to have achieved a complete metabolic response (CMR) (Figure 3).

No clinical or laboratory evidence of PTLD recurrence was observed during the follow-up. Renal graft function has remained stable (eGFR 68.9 mL/min/1.73 m²), and islet graft function has been preserved with a fasting C-peptide level of 0.61 ng/mL (Figure 3).

## Discussion

Islet transplantation is a minimally invasive therapeutic option for insulin-dependent diabetes mellitus, which involves the isolation and transplantation of islets from donor pancreata. Since the introduction of the “Edmonton Protocol” in 2000 [3], the number of clinical cases has increased globally, with continual improvements in islet isolation techniques [4] and immunosuppressive regimens [5] contributing to better outcomes [6,7]. The first human islet transplant in Japan was performed in 2004. A multicenter clinical trial under the Advanced Medical Care B program was launched in 2013 with the aim of achieving national insurance coverage. Based on these outcomes [8], allogeneic islet transplantation was officially approved and covered by the national insurance system in April 2020. Since then, the number of islet transplantation procedures performed in Japan has gradually increased.

The incidence of PTLD varies depending on the type of solid organ transplanted and intensity of immunosuppressive therapy required. In general, transplants that require more potent immunosuppression, such as heart, lung, pancreas, and small intestine transplants, are associated with higher PTLD incidence rates ranging from 10% to 25%. In contrast, liver and kidney transplants, which typically involve less intensive immunosuppression, have lower incidence rates of approximately 1%-5% [9]. One of the major mechanisms of PTLD development is uncontrolled B-cell proliferation because of the reactivation of EBV due to excessive immunosuppression [10]. EBV infection, particularly in EBV-seronegative recipients receiving grafts from EBV-seropositive donors, is a well-established risk factor for PTLD. Therefore, assessment of both donor and recipient EBV serostatus is strongly recommended when considering transplantation. In the present case, however, evaluation of the donor's EBV serostatus was not performed, and it is recommended that such evaluation be added in high-risk cases. Immunological tolerance is often achieved in hematopoietic stem cell transplantation and other forms of cellular therapy, thereby reducing the long-term requirement for immunosuppressive treatment and consequently leading to a lower incidence of PTLD [2]. Although islet transplantation is a form of cellular transplantation, paradoxically, it requires potent immunosuppression, similar to that used in whole-organ pancreas transplantation. Moreover, repeated islet infusions are often necessary, requiring multiple courses of strong induction therapy with agents such as ATG or basiliximab. Therefore, the cumulative immunosuppressive burden associated with multiple transplants may significantly increase the risk of developing PTLD. In the present case, in addition to islet transplantation, the patient had previously undergone both living-donor kidney transplantation and deceased-donor pancreas transplantation, which necessitated long-term immunosuppressive therapy. Extended exposure is a major risk factor for the development of PTLD.

According to the National Comprehensive Cancer Network (NCCN) guidelines, treatment strategies for PTLD are determined based on histological subtype and EBV status, with a fundamental emphasis on the reduction of immunosuppression (RIS). In early lesions, the earliest and least aggressive forms of PTLD, rituximab (RTX) monotherapy has demonstrated a response rate exceeding 70%. However, in monomorphic PTLD, which is more aggressive, the response rate to RTX monotherapy declines to approximately 40-60% [11]. For monomorphic subtypes, advanced-stage disease, RTX-refractory cases, EBV-negative PTLD, or those with a high tumor burden, combination chemotherapy in addition to RIS is recommended (Table 2) [12]. In the present case, the patient was diagnosed with monomorphic PTLD of the DLBCL subtype, which was CD20-positive and EBER-negative. Considering the aggressive disease course, systemic chemotherapy was initiated with R-CHOP in addition to RIS. After four cycles, the treatment response was evaluated as PR. Therefore, salvage chemotherapy with DA-EPOCH-R was administered, which resulted in CMR.

**Table 2:** PTLD treatment strategy (based on 2023 NCCN guidelines). ABVD, adriamycin (doxorubicin), bleomycin, vinblastine, and dacarbazine; DA-EPOCH-R, dose-adjusted etoposide, prednisone, vincristine, cyclophosphamide, doxorubicin, rituximab; DLBCL, diffuse large B-cell lymphoma; EBV, Epstein-Barr virus; NCCN, National Comprehensive Cancer Network; PTLD, post-transplant lympho- proliferative disorders; R-CHOP, rituximab, cyclophosphamide, hydroxydaunorubicin (doxorubicin), oncovin (vincristine), prednisone; RIS, reduction of immunosuppression; RTX, rituximab

Pathology Type	EBV Status	Treatment
Early Lesion	Positive	RIS
Polymorphic PTLD	Positive	RIS + RTX monotherapy
Monomorphic PTLD (e.g., DLBCL)	Positive	RIS + RTX monotherapy
	Negative	RIS followed by upfront chemotherapy (e.g., R-CHOP or DA-EPOCH-R)
Hodgkin-like PTLD	Positive/Negative	RIS followed by chemotherapy (e.g., ABVD)

A considerable number of islet transplantation cases, including islet transplantation alone and islet transplantation following kidney transplantation, have been reported. However, PTLD following islet transplantation remains extremely rare. A PubMed search using the terms “islet transplantation” and “PTLD” identified only two previously reported cases worldwide, with no reports originating from Japan [13]. In both cases, immunosuppressive therapy was discontinued and multimodal treatments were administered; however, graft function was ultimately lost in both instances (Table 3). In contrast, in the present case, careful modulation of immunosuppressive therapy allowed the preservation of graft function. Rather than complete discontinuation of tacrolimus, trough levels gradually decreased within a controlled range. This strategy led to successful maintenance of islet graft function. Meanwhile, in reports of PTLD following other solid organ transplants such as pancreas or kidney transplantation, a treatment approach involving reduction of immunosuppression combined with RTX-based chemotherapy has been associated with higher rates of graft function preservation. For instance, one study on pancreas transplant recipients reported a three-year graft survival rate of 77% following this strategy [14], while kidney transplant recipients have also shown favorable outcomes, with many maintaining stable graft function under similar therapeutic adjustments [15]. These findings provide valuable insights into the management of PTLD following islet transplantation and suggest a potential approach for balancing effective lymphoma treatment with long-term graft preservation.

**Table 3:** PTLD after islet transplantation cases Summary of previously reported cases of PTLD after islet transplantation, including the current case. Modified from reference [13]. DLBCL, diffuse large B-cell lymphoma; DNA, deoxyribonucleic acid; EBV, Epstein-Barr virus; IAK, islet transplantation after kidney transplantation; ITA, islet transplantation alone; MTX, methotrexate; PTLD, post-transplant lymphoproliferative disorders; R-CHOP, rituximab, cyclophosphamide, hydroxydaunorubicin (doxorubicin), oncovin (vincristine), prednisone; Tx, transplantation

	Case 1 [13]	Case 2 [13]	This case
Age/Sex	44/male	58/female	55/female
Date: After 1^st^ Tx	80months	2years	65months
Tx type	ITA	ITA	IAK
EBV-DNA	negative	500>(copy/μgDNA)	230(copy/μgDNA)
Pathology	DLBCL	DLBCL	DLBCL
Treament	Surgery+MTX	R-CHOP	R-CHOP
Immuno-suppression	discontinued	discontinued	tapered
Graft function	Failed	Failed	Maintained

Islet transplantation is a minimally invasive procedure that requires advanced immune regulation, and improvement in long-term outcomes depends on the proper management of post-transplant complications. This case report presents an extremely rare instance of PTLD following islet transplantation and provides valuable insights by demonstrating the successful treatment of PTLD while maintaining graft function. These findings contribute to our understanding of the future development of islet transplantation. Individualization of immunosuppressive therapy and early risk management are key factors that are likely to lead to further success in islet transplantation.

## Conclusions

Herein, we report a case of PTLD following islet transplantation. By continuing immunosuppressive therapy and administering chemotherapy, we were able to treat the patient’s condition without abolishing graft function. This finding provides important insights for future treatment guidelines for PTLD after islet transplantation.
